# Towards Automating Personal Exercise Assessment and Guidance with Affordable Mobile Technology

**DOI:** 10.3390/s24072037

**Published:** 2024-03-22

**Authors:** Maria Sideridou, Evangelia Kouidi, Vassilia Hatzitaki, Ioanna Chouvarda

**Affiliations:** 1 Lab of Computing, Medical Informatics, and Biomedical-Imaging Technologies, School of Medicine, Aristotle University of Thessaloniki, 54124 Thessaloniki, Greece; 2 School of Physical Education and Sport Science, Aristotle University of Thessaloniki, 54124 Thessaloniki, Greece; kouidi@phed.auth.gr (E.K.); vaso1@phed.auth.gr (V.H.)

**Keywords:** pose estimation models, BlazePose, real-time biomechanical feedback-(BMF), kinematics, machine learning, signal processing, computer vision, virtual coach, elderly

## Abstract

Physical activity (PA) offers many benefits for human health. However, beginners often feel discouraged when introduced to basic exercise routines. Due to lack of experience and personal guidance, they might abandon efforts or experience musculoskeletal injuries. Additionally, due to phenomena such as pandemics and limited access to supervised exercise spaces, especially for the elderly, the need to develop personalized systems has become apparent. In this work, we develop a monitored physical exercise system that offers real-time guidance and recommendations during exercise, designed to assist users in their home environment. For this purpose, we used posture estimation interfaces that recognize body movement using a computer or smartphone camera. The chosen pose estimation model was BlazePose. Machine learning and signal processing techniques were used to identify the exercise currently being performed. The performances of three machine learning classifiers were evaluated for the exercise recognition task, achieving test-set accuracy between 94.76% and 100%. The research methodology included kinematic analysis (KA) of five selected exercises and statistical studies on performance and range of motion (ROM), which enabled the identification of deviations from the expected exercise execution to support guidance. To this end, data was collected from 57 volunteers, contributing to a comprehensive understanding of exercise performance. By leveraging the capabilities of the BlazePose model, an interactive tool for patients is proposed that could support rehabilitation programs remotely.

## 1. Introduction

The maintenance of functional capacity in the elderly stands as a crucial public health priority [1]. Physical activity (PA) offers many benefits for human health for all age groups. Particularly for older adults, regular exercise is correlated with decreased risks of cognitive decline, depression, dementia, obesity, diabetes, and cardiovascular diseases [2,3]. Furthermore, it aids in enhancing muscle strength, preserving movement coordination, improving balance and flexibility, and preventing falls. All these factors contribute to healthy aging and enable older adults to be self-sufficient and maintain their autonomy [4].

Despite the benefits of PA, most of the elderly population does not exercise. Fear of falling and musculoskeletal injuries is a common concern that leads older adults to avoid physical activities that they are still able to perform [5]. In addition to these fears, the recent COVID-19 pandemic or inaccessibility to facilities may affect their confidence to leave home and attend classes in the community. Tele-exercise (TE) systems can override these limitations by allowing the users to exercise safely in their home environment.

TE systems lie in the field of real-time biomechanical feedback (BMF). In the last decade, advanced technologies have been widely used to enhance motion skills in sports and accelerate the rehabilitation process. BMF is a method of therapy and control of the human body that uses sensors to measure body functions and activities that cannot be perceived by human senses. The collected data are analysed and returned to the subject through visual, auditory, or other stimuli. The subject responds by modifying its activity based on instructions, thus closing the feedback loop. When implementing BMF systems to support physical exercise routines, real-time guidance is the optimal choice. 

Range of motion (ROM) testing and kinematic analysis (KA) of exercise are essential for evaluating and studying PA. ROM testing refers to the measurement and evaluation of the movement capabilities of specific joints or body parts, while KA is a powerful method for objective assessment of PA in a three-dimensional (3D) space [6,7]. For years, the assessment of trainees’ performance was conducted through visual inspection by the trainers, while joint ROM was measured with instruments such as goniometers and inclinometers. This approach disrupted the continuity of the exercise and rendered the trainer’s participation necessary for the data collection process. 

Sensor systems such as inertial measurement units (IMUs) and video-based human pose estimation (HPE) technologies enable the digital collection and KA of exercise data and the continuous monitoring of the trainee without interruptions [8]. In 2013, Toshev et al. presented DeepPose, a model for HPE based on deep neural networks [9]. The integration of deep learning algorithms into the architecture of HPE models has provided improved accuracy and reduced computational demands, enabling the simultaneous assessment of exercise alongside data collection. In 2020, Bazarevsky et al. proposed BlazePose, a lightweight and fast HPE model built on a convolutional neural network architecture [10]. Designed for fitness applications, it substantially enhances motion detection rates. In contrast to earlier HPE technology, such as the OpenPose model [11], BlazePose exhibits a significant speed improvement up to 75 times on a single mid-tier phone CPU, all while scaling to a larger number of keypoints and providing 3D support.

Artificial intelligence (AI) techniques, such as machine learning (ML) and deep learning, are found to increase the accuracy of processing human activities in real-time [12]. Two-dimensional and multi-layered convolutional neural network models are used for automatic recognition and classification of human actions and optimal human activity recognition (HAR) analysis [13]. Nowadays, high-level features (HLF) are preferred to increase HAR performance. HLFs are proven to be accurate and effective in the combination of multiple datasets, comparison, and classification of different activities, which is of high importance for HAR and HA description [14]. In 2021, Liu et al. presented an innovative modeling technique that partitions human activity into motion units (MU), akin to phonemes in speech recognition, showcasing comparable accuracy with fewer parameters, enhanced separability, and remarkable scalability across datasets, facilitating interpretable, extensible, and scalable HAR [15].

In 2023, Fabbrizio et al. provided an overview of the evolving landscape of telehealth, emphasizing the opportunities for future research [16]. This encompasses the creation of tailored AI algorithms for analysing data derived from Internet of Things (IoT) devices, leading to the provision of personalized recommendations for users. Additionally, there is a focus on minimizing the risk of device failures and malfunctions in TE equipment. Concurrently, research should be conducted to identify and address barriers hindering the adoption of smart devices and ΤΕ services to enhance access to the elderly population.

Based on previous research on TE approaches, we set the goals for our approach. The aim of the present study was to analyse the PA performance of patients and older adults and develop a novel, user-friendly assisted TE system with specific properties. Our proposal requires only a computer camera or smartphone as a prerequisite and uses the BlazePose model. We intended to offer a low-budget solution utilising a posture estimation model. Next, we aimed to design a TE system suitable for spontaneous use. In this context, we intended to integrate an exercise recognition system. This was necessary, especially since our system is designed to assist older adults who may face challenges remembering or interpreting instructions correctly. Our design offers multiple user features, including exercise recognition, repetition counting, and real-time guidance.

## 2. Materials and Methods

### 2.1. Real-Time TE System: Utilising Pose Estimation Technology

In the literature, there is a plethora of HPE models with varying properties. The choice of the most suitable model depends on the application’s requirements. Our selection criteria focused on single-person recognition, the number of detected body keypoints, returned coordinates for each keypoint, computational power requirements, and the detection rate. The model that effectively meets the requirements of our approach is BlazePose [10].

**Figure 1:**
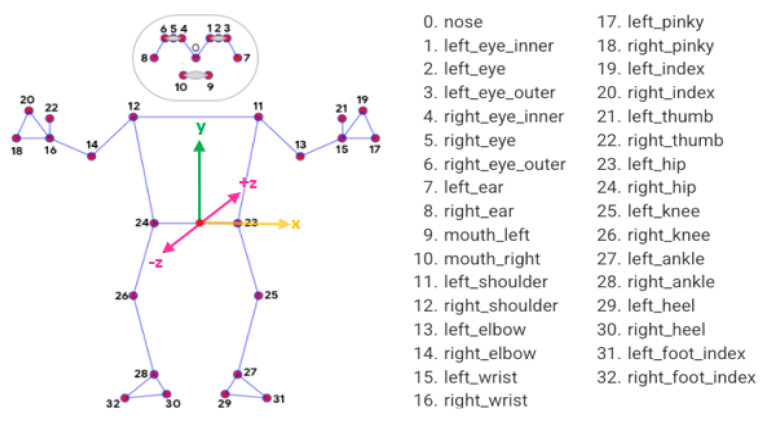
Human body keypoint topology map and coordinate systems of BlazePose.

The BlazePose model outperformed others by returning the most keypoints, and its lightweight nature made it suitable for applications on Android and websites. Since the objective was to track human movement in real-time, it was deemed necessary to choose a computationally efficient model, compensating for the need for extremely accurate point prediction. BlazePose operates at a rate of up to 30 frames per second on smartphones, takes RGB frames from a video or web camera as an input, and produces 33 body keypoints for a single person in each frame, as shown in Figure 1.

### 2.2. Experimental Protocol

Volunteers were asked to perform five simple physical exercises, with five or ten repetitions per exercise, at a self-selected speed. For both groups, we performed kinematic analysis (KA) on the exercises and statistical studies on the KA findings and we investigated overall kinematic changes while considering anthropometric factors. To elaborate further, we performed ROM testing and gathered the most common errors that occurred during exercise. Using these guidelines, we evaluated patients’ performance to determine whether they followed the prescribed form or deviated from it.

#### 2.2.1. Study Population

For our research, we collected video-type data from 57 volunteers, of whom 11 were patients or older adults and 46 were trainers. Fifty-seven volunteers participated, consisting of 35 females and 22 males. The patients were enrolled in rehabilitation programs across several municipal gyms in Thessaloniki. The trainers’ performance was used as a model for the development of our system. Based on exercise performance, the study group was divided into two samples: (a) optimum performance and (b) under evaluation performance/patients’ performance. For the trainers’ group, we retained only the repetitions performed correctly, considering deviations within a 10–20% error margin.

For every participant, we documented the following information: gender, age, weight, height, PA experience, and medical history. Additionally, we calculated the body mass index (BMI). Notably, the dominant side of the body was not recorded. For the study’s purposes, we presumed the right limb to be dominant, as is typically the case. Regarding the health of the patient group, most of them were older adults and suffered from diseases which are summarized as heart diseases, kidney diseases, and neurological and musculoskeletal disorders. It should be noted that most patients were familiar with exercise, they just needed supervision due to their age and health condition. For additional information regarding statistical studies on the study population, please refer to Appendix A.

The study was conducted in accordance with the Declaration of Helsinki and approved by the Research Ethics and Ethics Committee (REC) of Aristotle University of Thessaloniki (protocol code 20005/2023 and date of approval 19 January 2023). Informed consent was obtained from all subjects involved in the study. Additionally, we added blur on the faces of the volunteers and removed the background sound from the videos.

#### 2.2.2. Recording Equipment

For the video recording, we used a Xiaomi Redmi Note 7 smartphone. The camera resolution was 1920 × 1080 pixels, with a video recording frame rate of 29 frames per second (fps). The video recording was conducted using the smartphone’s rear-facing camera system. The smartphone was kept in a fixed position at an approximate height of 1.40 m from the ground, and the recording distance from each volunteer ranged between 4.5 m and 5 m. No tripod was utilised throughout the recording process.

During the second phase, following the development of our TE system, we conducted real-time testing and scenario-based experiments using a web camera set at 640 × 480 pixels resolution and recording at a frame rate of 30 fps.

#### 2.2.3. Exercise Repertoire

**Figure 2:**
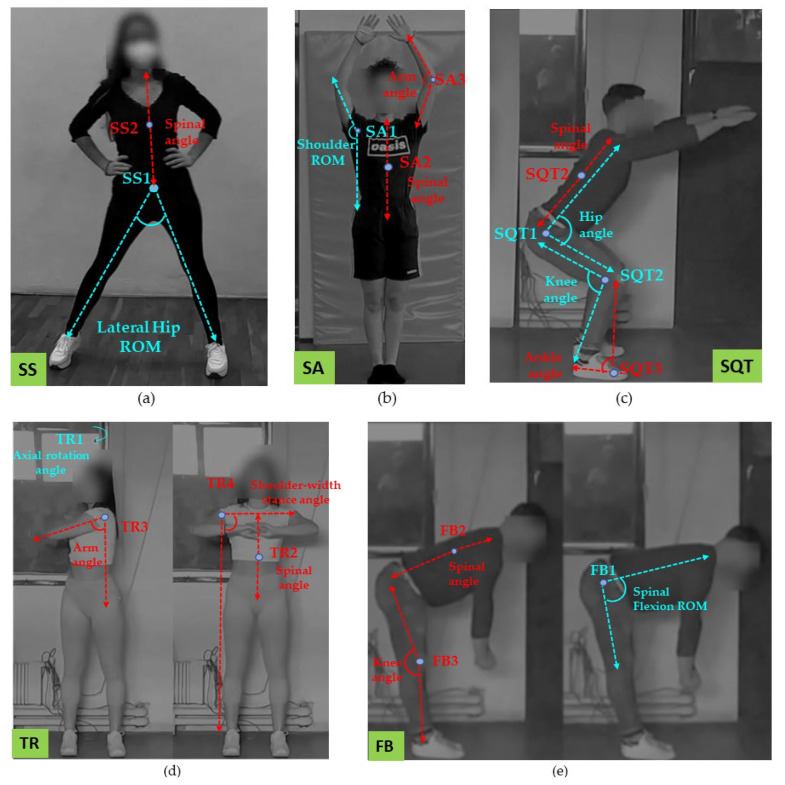
Kinematic analysis (KA) of the exercise repertoire. The first row includes (**a**) SS, (**b**) SA, and (**c**) SQT while the row below contains (**d**) TR and (**e**) FB. We mark the guidelines that involved inspection for execution errors in red, while we mark the studied angles for the ROM testing in blue.

The volunteers performed a set of five exercises, depicted in Figure 2, that focused on training the upper limbs, lower limbs, and trunk while assessing balance and flexibility. The exercise repertoire consisted of side steps (SS) for warming up, shoulder abductions (SA) for arm rehabilitation, trunk rotation (TR) for trunk muscle strengthening and balance, and standing forward bends (FB) for flexibility and mobility improvement. Detailed evaluations were conducted, focusing on the ROM for the hips, shoulders, and trunk, as well as movement coordination and trajectory monitoring. The routine was carefully selected, with attention to its suitability for patients and older adults. Details regarding the instructions we gave to the volunteers and the ROM analysis ROM per exercise are shown in Table 1.

In SS, SA, and TR exercises, the user faced the camera. Due to positioning, the spinal alignment check was assessed in the YZ plane. However, limitations in BlazePose’s z component estimation render YZ measurements unreliable. As a result, our focus was directed towards examining feasible angles.

### 2.3. Kinematics

KA describes the movements of the body through space and time, including linear and angular displacements, velocities, and accelerations [7]. In our study, we applied KA principles to assess various key factors, including (a) ROM and execution error detection, (b) exercise velocity, and (c) movement coordination.

As shown in Figure 2, to suit the parameters of the KA we associated the guidelines with corresponding joint angles on the body. Note that BlazePose provides trajectory data. For the transition from trajectories to angle time series, we used the cosine formula. We obtained ROM data by analysing the angle time series, focusing specifically on local maxima. These local maxima represented the points where the joint or body part achieved the highest angular positions. In assessing the overall performance of each participant, we determined the median of the maximum angles attained across each exercise. This calculation provides a robust measure, capturing the central tendency of the highest angular positions achieved during all repetitions. Additionally, we consulted the literature to compare our KA findings with standard ROM values. The normative values were provided by the American Academy of Orthopaedic Surgeons (AAOS), and they are goniometer measurements [17].

For the angular velocity calculation, we utilised the following formula:

(1) $$\omega \left(t\right)=\frac{d\theta }{dt},$$

where dt corresponds to the duration of one repetition and dθ represents the inscribed angle. This analysis allows us to quantify the rate of change in angular position over time, providing valuable insights into the rotation dynamics of the observed motion. We studied the patients’ motion coordination by monitoring the participation order of N body parts. The task was handled algorithmically as a problem of phase shift computation among N time series using cross-correlation. This approach allows the quantitative assessment of the temporal relationships and sequencing of movements across N predefined body parts.

Finally, statistical analysis was conducted to investigate potential differences in PA performance (ROM and execution errors) among patients and trainers and females and males. An independent *t*-test was used to compare dependent variables based on each group (female trainers (FT), female patients (FP), male trainers (MT), and male patients (MP)). For all statistical analyses, the type I error rate was set at 0.05. To eliminate potential confounding influences, we incorporated age, height, and BMI as covariates. The examination of all continuous dependent variables was conducted through the application of the general linear model.

### 2.4. Data Processing

**Figure 3:**
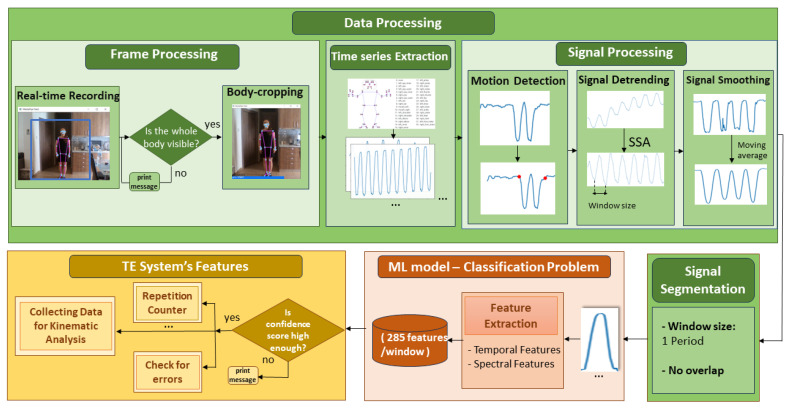
The overview of the analysis performed for the development of our tele-exercise (TE) system using signals derived from BlazePose. The three rectangles refer to the following sections: Section 2.4, which details the data processing; Section 2.5, which describes the classification problem; and Section 3.3, which introduces the basic facilities of the TE system.

As shown in Figure 3, the preprocessing of the collected data includes three steps: (a) frame processing, (b) applying the BlazePose architecture to convert image data into signal data, and (c) time series processing.

#### 2.4.1. Body-Centering Frame Processing

Frame processing involves isolating the human pose through a body-cropping process. To ensure an efficient pose estimation, unaffected by the individual’s distance from the recording equipment and camera resolution, we developed a body-cropping algorithm. Moreover, it has been reported that these algorithms enhance the accuracy of HPE models without any modification to their network architecture [18]. The primary objective was to establish a personalized cropping frame centered on the body of each individual. Utilising BlazePose references, the midpoint between the hips was reported as the center of the body. This point was chosen as the center of the cropping frame. In addition, a reference distance (RDS) was selected for each user based on their arms’ dimensions. The Euclidean distance between the shoulder and the index finger was calculated. We applied the same methodology for both left and right arms, and the average of the arms’ lengths determined the RDS. Based on the center point and the RDS, we calculated the cropping borders and reshaped the frames.

After isolating the human figure, the BlazePose architecture was applied to the reshaped frames. As mentioned above, the BlazePose model takes a frame as its input and outputs a vector of 33 3D body keypoints. By iteratively reconstructing the generated points for multiple frames, we extracted 99 time series representing limb movements for each trial. The following section describes the processing techniques applied to the extracted signals.

#### 2.4.2. Motion Time Series Processing

The time series processing section includes (a) a motion detection algorithm, (b) removing trends that appeared due to measurement errors, (c) smoothing the extracted time series, and (d) signal segmentation.

We developed a motion detection algorithm to define the beginning and the end of each exercise and to separate exercise phases from pauses. We computed the percentage amplitude modulation between consecutive samples of a signal. If this modulation surpassed a default threshold, the algorithm identified body activity. The amplitude threshold was empirically set at 30%. We used the same methodology to detect pauses and the end of the execution.

A crucial step in the time series processing involved the removal of an unnecessary trend in the data. As mentioned, during video recording, a tripod was not utilised. Consequently, minor and random adjustments of the hand holding the recording equipment were reflected in the data. Detrending was accomplished with the use of the singular spectrum analysis (SSA) [19] decomposition method, which makes no assumptions about the nature of the time series. We used SSA to decompose every signal into a set of 12 summable components that are reconstructed into three grouped time series referred to as the trend, periodicity, and noise. For our analysis, the task was simplified to detect only the trend component. Once the trend component was reconstructed, it was subtracted from the original signal, and we obtained the detrended time series. The detrending process is necessary only if the recording device is not placed in a fixed position. However, this method of use is not recommended.

To address data noise and smooth our time series, we applied the moving average (MA) technique. This method involves replacing a data point with the average of neighboring values within a moving window. Operating as a low-pass filter, a moving average effectively eliminates short-term fluctuations to emphasize a more profound underlying trend. The mathematical definition of the MA is as follows:

(2) $$\bar{x_{i}}=\frac{1}{2Μ+1}\sum_{j=-M}^{j=M}x[i+j],$$

where the average data point is calculated by averaging the points contained in a moving window with size 2M and centered around $x_{i}$.

For the segmentation of the signals, we followed a repetition-based approach. We calculated the period for each signal, which was equivalent to the duration of an exercise repetition. Leveraging this information, we performed the signal segmentation. The resulting dataset, structured based on repetitions, contained a total of 1811 samples.

### 2.5. Exercise Recognition as a Classification Problem

**Figure 4:**
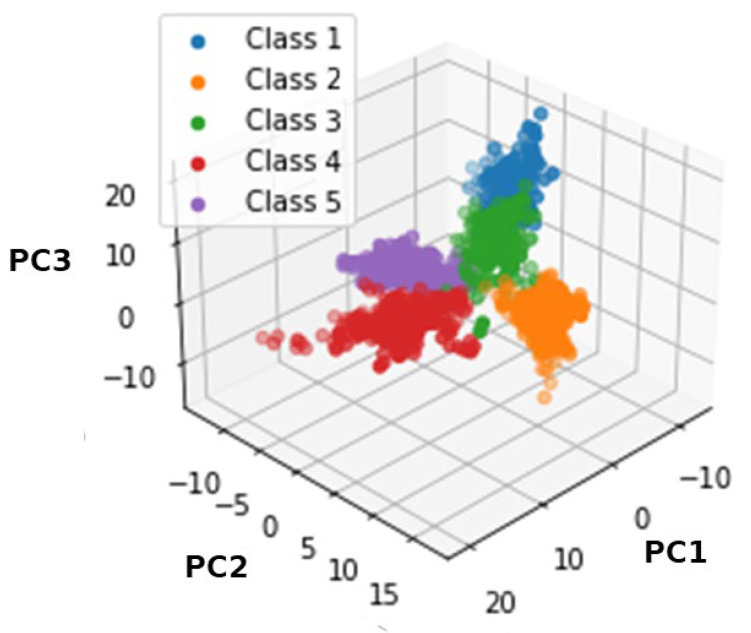
Visualization of the classification problem using the three first principal component analysis (PCA) components. Class 1 corresponds to the SS, class 2 corresponds to the SA, class 3 corresponds to the TR, class 4 corresponds to the FB, and class 5 corresponds to the SQT exercise.

As previously mentioned, we aimed to integrate an exercise recognition system into our TE system. The classification problem involves assigning each exercise repetition to one of the five exercises in our repertoire, utilising a sequence of kinematic data as the input. We approached the classification task using supervised ML methods. The problem comprises five classes corresponding to the introduced exercises. Figure 4 highlights the dataset’s separability. The dataset contains a total of 1811 records. Class 1 corresponds to the SS exercise and consists of 177 samples. Class 2 corresponds to the SA exercise and consists of 459 samples. Class 3 corresponds to the TR exercise and consists of 257 samples. Class 4 corresponds to the FB exercise and consists of 438 samples. Class 5 corresponds to the SQT exercise and consists of 480 samples.

#### 2.5.1. Feature Extraction

For the model training feature set, we considered twelve time domain features (mean, maximum, minimum, root mean square (RMS), variance, standard deviation, crest factor, kurtosis, and skewness) and seven frequency domain features (mean and max of band power spectrum, sum of total band power, standard deviation, peak, kurtosis, and skewness of band power). In total, we evaluated 19 features per signal. Each instance consisted of 33 kinematic time series for each coordinate (x, y, and z). To simplify the analysis, we narrowed down the examined body keypoints to five basic points, located on the torso, left and right arms, and left and right legs. The set of 19 features was eventually extracted for 15 time series. Consequently, the final size of the feature vector was 285 for each instance.

In addition, we formed a feature vector obtained with a principal component analysis (PCA). The feature space was reduced by retaining the first 22 principal components, which collectively explained 90% of the total variance in the data. This step aimed to capture the most salient information while reducing the dimensionality of the dataset.

#### 2.5.2. Model Training and Evaluation

Various classification algorithms, such as random forest (RF) [20], support vector machine (SVM) [21], and decision trees (DT) [22], were explored to address the classification problem. We evaluated three different kernel mappings: linear (${SVM}_{L}$), polynomial (${SVM}_{P}$), and radial basis function (${SVM}_{RBF}$). DT was included as a baseline method, keeping in mind the overfitting problem of the algorithm [20].

The dataset of 285 and 1811 samples was divided into a training set, comprising 70% of the total records, and a test set, comprising the remaining 30%. Notably, the test set consisted exclusively of unseen volunteers’ data, ensuring that the model performance was evaluated on instances not encountered during the training phase. In particular, we reserved data from 17 out of the 57 volunteers to create the test set.

Ten-fold cross-validation was used to assess the performance and generalizability of the predictive model [23]. For the classifiers’ performance comparison, we examined several metrics for the test set, including accuracy, precision, sensitivity, F1 score, and specificity. In addition, the training and inference time was calculated for each model. The selection of the preferred ML algorithm relied primarily on accuracy, with due consideration given to the other metrics as well.

## 3. Results

In this section, we describe and discuss the results from the statistical analysis of ROM and the assessment of execution errors. The classifiers’ performance was evaluated, and we selected the optimal algorithm for our classification problem. Our discussion also encompasses the assessment of the reliability of the BlazePose model based on the insights garnered from our user experience. Moreover, we present our TE system’s functionalities and provide an illustrative case scenario, showcasing the operation of the entire system.

### 3.1. Kinematic Analysis Results

#### 3.1.1. Range of Motion Testing Results

**Figure 5:**
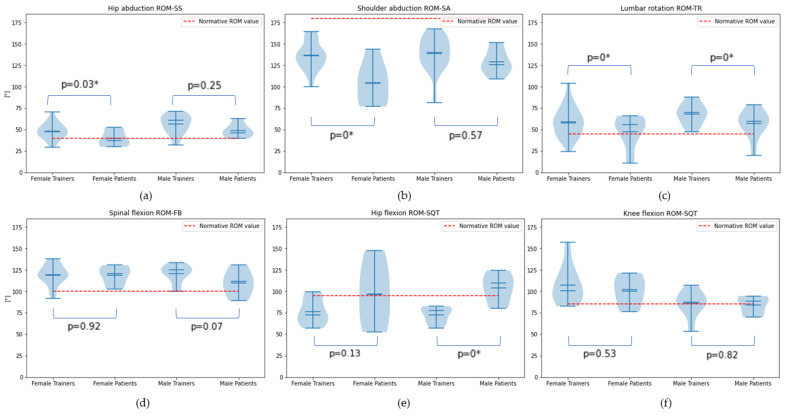
Violin plot representations of the study population’s ROM, performance comparison between patients and trainers, and visualization of the normal ROM values. Testing: (**a**) hip abduction ROM for SS, (**b**) shoulder abduction ROM for SA, (**c**) lumbar rotation ROM for TR, (**d**) spinal flexion ROM for FB, and (**e**,**f**) hip and knee flexion ROM for SQT exercise. We mark the normative ROM values in red. The statistically significant differences are marked with an asterisk (*).

It is evident from Figure 5 that in the KA of our exercise repertoire, a consistent pattern emerged, showcasing that trainers exhibited an increased ROM compared to patients, especially in the SS, SA, TR, and SQT exercises where the detected differences were considered statistically significant. Analysis of sex differences in angular motion indicates that the MT group consistently displayed a greater overall ROM compared to the FT group. This difference was particularly evident in the SS and SQT exercises, where statistical significance was observed between sexes. For the patient group, males likewise demonstrated superior ROM performance, albeit without reaching statistical significance.

We correlated the findings of the KA with anthropomorphic factors of the study population. There was a reduction in ROM attributed to age and BMI within our sample. In the TR exercise, a notable linear correlation emerged between lumbar rotation ROM, age (linear regression score = −0.77, *p* = 0), and BMI (linear regression score = −0.59, *p* = 0). In the FB exercise, a notable linear correlation was detected between trunk forward flexion ROM, age (linear regression score = −0.68, *p* = 0), and BMI (linear regression score = −0.62, *p* = 0).

During the SS exercise, 59.01% of the participants exhibited increased ROM on the dominant side, and 67.50% did during the TR exercise. Contrastingly, 52.63% of participants displayed greater ROM on the non-dominant side during the SA exercise.

Finally, potential differences in motions’ angular velocity were investigated, comparing patient and trainer groups. MP appeared to perform with increased velocity (1.03 ± 0.19 vs. 0.52 ± 0.25 rad/s) only in the SQT exercise compared to MT, with a statistically significant difference (t = 3.44, *p* = 0). In the SS and SA exercises, the trainers exhibited higher velocities compared to the patients. However, in the TR, FB, and SQT exercises, patients executed the movements hastily.

#### 3.1.2. BlazePose Model’s Reliability

The normal hip abduction ROM value is stated as 40° according to the AAOS. In this study, the average hip abduction ROM values were between 40.37° and 55.50°. As shown in Figure 5, the hip abduction ROM values obtained in this study were found to be similar to the reference values. The normative value for shoulder abductions is 180°. In the present study, the average shoulder abduction ROM values were between 146.38° and 165.10° and were found to be lower than the reference sources. Normative lumbar rotation values are stated as 45°. In the present study, the rotation ROM values were between 51.65° and 70.19°, higher than the reference values. The normal value for spinal flexion ROM is stated as 100°. The current study average values were between 109.40° and 120.85° degrees, similar to the reference values. The normative values for hip and knee flexion while squatting are 95° and 85°, respectively, according to Hemmerich et al. [24]. In the present study, the average values were between 72.63° and 103.62° for hip flexion and between 83.97° and 106.90° for knee flexion and were found to be similar to the reference values.

#### 3.1.3. Performance Evaluation

**Figure 6:**
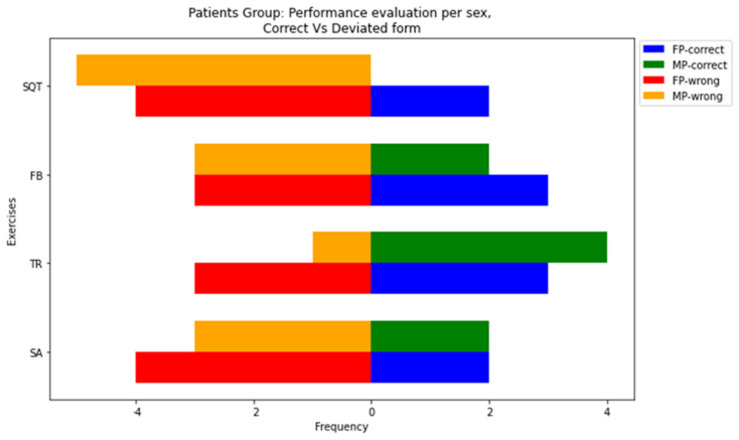
Patient performance evaluation by sex. Female patients who performed without and with execution errors are represented by blue and red, respectively, while male patients who performed without and with errors are represented by green and orange colors, respectively.

It is evident from Figure 6 that patients’ performance deviated significantly from the prescribed form. Studying the SA exercise, 63.63% of patients demonstrated significant performance alterations. A significant proportion, specifically 36.36%, within the patient group deviated from the guideline of keeping the arms vertical to the body axis during TR. In the patient group, 54.54% performed TR by moving their head, trunk, and arms as a single unit, unlike trainers. In the FB exercise, 54.54% of patients bent their legs beyond the acceptable tolerance. Regarding the SQT exercise and the proper positioning between knees and toes, we noted significant deviations for 81.81% of the patients.

### 3.2. ML Algorithm Performance

**Figure 7:**
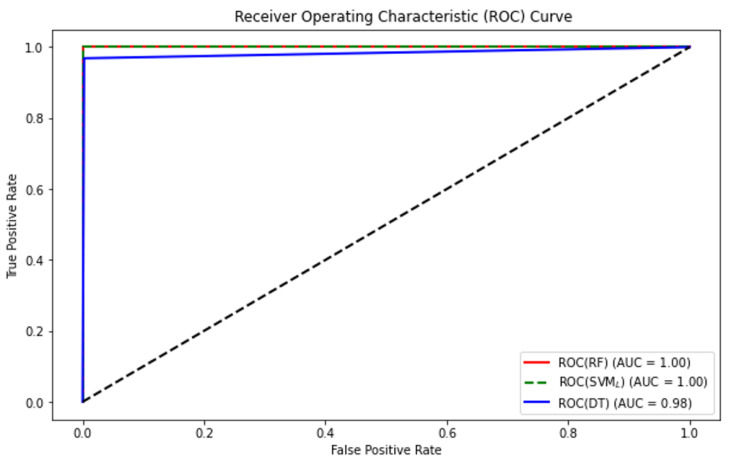
Receiver operating characteristic (ROC) curve and AUC scores of the examined classifiers.

**Figure 8:**
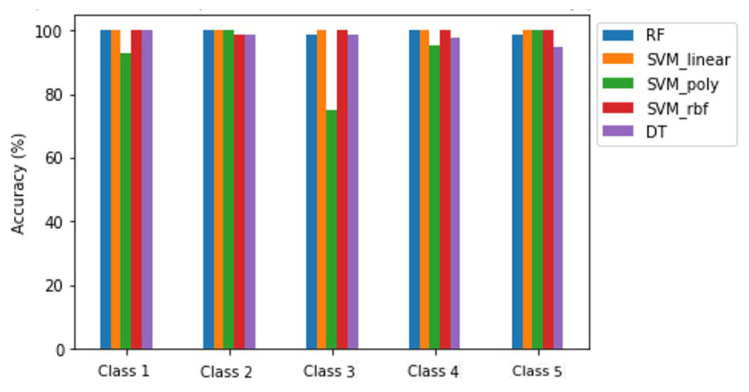
Test set accuracy per class for the examined classifiers.

As shown in Table 2, after a meticulous evaluation, the RF classifier outperformed due to its ability to achieve high test set accuracy (100%). However, concerns regarding overfitting and computational efficiency prompted the integration of PCA into the workflow. Post-PCA integration, we re-evaluated the classifiers’ performance. We observed a marginal decrease in RF’s accuracy (99.63%) and an increase in the ${SVM}_{L}$ classifier’s accuracy. Notably, after PCA application, SVM’s accuracy increased to 100%. With this result, the data can be considered linearly separable. Τhis finding can be easily observed in Figure 4. As evidenced by the high area under the ROC curve (AUC = 1.00) in Figure 7, our ML model has strong discriminatory power and performs well across different decision thresholds. We opted for the ${SVM}_{L}$ classifier, achieving a remarkable accuracy of 100% for each class as displayed in Figure 8. The computational efficiency gains achieved through PCA were evident, enabling faster model training and inference contributing to an improved response rate and overall performance of our real-time TE system.

### 3.3. TE System’s Facilities

**Figure 9:**
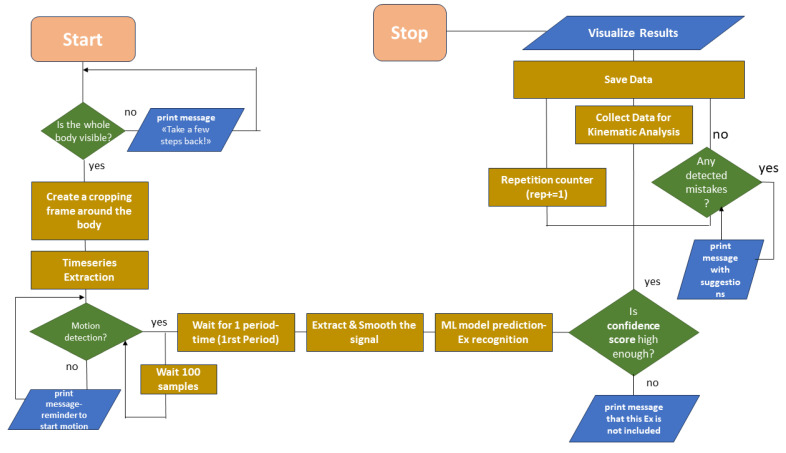
Simplified flowchart of all the operations and controls performed by the TE system.

Based on our findings, we developed a cutting-edge TE system designed to offer users meticulous guidance through visual feedback messages on their screens. As presented in Figure 9, we included several functionalities such as exercise recognition, repetition counting, inactivity and change in exercise detection, correction of execution errors, and ROM data analysis.

The system can operate on either a computer or a smartphone. In any case, we recommend that the recording device be placed in a fixed position. This ensures a comfortable user experience while avoiding alterations in the data. If opting for the smartphone version, the front-facing camera system is employed. This way the users can observe themselves and access the displayed feedback messages while exercising.

### 3.4. Real-Time Testing Scenario

**Figure 10:**
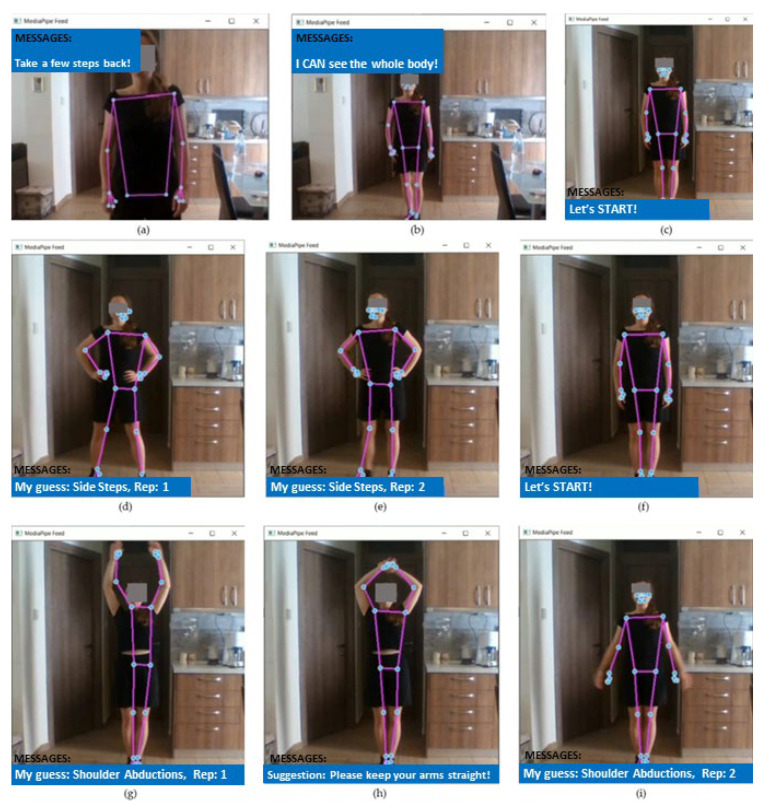
The TE system’s demonstration for the real-time testing scenario: (**a**) state: user’s body is not fully visible; (**b**) state: user’s body is fully visible; (**c**) state: user is inactive; (**d**) state: first exercise recognition, SS; (**e**) repetition counter initiation; (**f**) state: user is inactive again; (**g**) state: second exercise recognition, SA; (**h**) state: SA exercise, execution error detected; and (**i**) state: SA exercise, improved performance. Each figure includes the actual exercise, the identified keypoints and skeleton, and a blue feedback ribbon on the bottom that includes the detected exercise and repetition and, when an error is identified, the suggestion for correction.

To validate our TE system’s functionalities, we designed and tested a realistic scenario, demonstrated in Figure 10, simulating real-time user interactions. We asked a volunteer to perform two exercises (SS and SA) from our repertoire. The volunteer, although following a script, was allowed to pause at an unknown time and make spontaneous execution errors. The scenario and the TE system’s responses are outlined below:

The user presses the start button. A message, on the screen, prompts her/him to step back until their entire body is visible in the web camera. After the user’s figure is fully detected, automatic zooming and cropping are performed around it. Until the user starts exercising, the system detects inactivity and motivates them to begin. After some time, corresponding to an execution period, the system correctly predicts the current exercise (SS) and initiates the repetition counter. The user performs two repetitions of SS without errors and then stops exercising. The system detects the user’s inactivity and returns, again, a motivation message on the screen. The user starts performing the second exercise and the system correctly identifies it as SA, resetting and restarting the repetition counter. During checks for execution errors, the system detects alterations (excessive arm bending) and displays a message on the screen instructing the user to “Keep the arms straight!”. The system continues to provide feedback until the user improves the performance.

## 4. Discussion

The results showed that the BlazePose model’s ROM values closely align with the widely accepted normative references of the AAOS. Comparative analysis confirms the reliability of BlazePose measurements when body parts move within the camera’s capture plane. However, measurements may lack reliability for movements executed without direct camera exposure. Proper positioning and sampling within the camera’s view can reduce estimation errors and optimize BlazePose’s potential.

In addition, BlazePose successfully identified differences in ROM, speed, and exercise quality related to age, gender, BMI, and dominance. Notably, there was a reduction in ROM attributed to age and BMI within our sample, an observation that aligns with documented factors causing ROM limitations in adults [25,26]. Although for the majority of exercises, no statistically significant differences were found regarding velocities, the conclusions that can be drawn are interesting. Increased speed in patient performance was associated with prioritizing speed over proper technique, leading to ineffective workouts and an elevated risk of musculoskeletal issues. The analysis of exercise quality showed significant deviations from the norm in the patient group, potentially simplifying perceived difficulty. This simplification creates a misleading impression of an increased ROM for the patient group which, however, is not meaningful to investigate when deviations in execution are detected. Movement coordination analysis of the TR exercise indicated synchronized head, trunk, and arm movements for 54.54% of the patient group. Our findings validate existing reports indicating that older adults commonly perform TR as a cohesive movement [27]. Overall, male patients outperformed females. In conclusion, the evaluation of patients’ performance emphasized the need for guidance during exercise to prevent injuries and ensure effective rehabilitation and fitness progress.

In our proposal, all the preprocessing algorithms are automatically executed. Importantly, this approach eliminates the need for any manual data inspection and enables the implementation of the algorithms into our TE system without requiring any modifications.

Regarding the assessment of the ML model’s robustness presented for the exercise recognition task, we conducted testing on new, unseen volunteers’ data. This testing was conducted under real-time conditions, employing recording equipment distinct from that used in the training set. The model demonstrated efficiency across various use-scenario experiments, thus highlighting its potential regarding generalization.

We highlight limitations in BlazePose model implementation. A strong correlation between recording equipment quality and BlazePose’s performance was detected, especially in the z component. The model’s reduced performance in precise calculations makes it unsuitable for certain applications. It is apparent that this deficiency also affects the overall functionality of our method as it is based on the correct recognition of the user’s body. However, for equipment with a recording rate higher than 29 fps, the phenomena of exercise tracking inability were rare, and all system functions performed smoothly.

Recent research has employed data-driven methodologies to estimate various biomechanical metrics and support exercise routines. The approach of García-de-Villa et al. combined exercise recognition and evaluation as a single task, utilizing data from four IMUs and achieving accuracies between 88.4% and 91.4% [28]. Despite the excellent performance, we avoided the use of a sensor system due to cost and simplicity considerations. Numerous research studies have employed ML models using BlazePose keypoint data to predict falls in real-time scenarios [29,30]. To our knowledge, the work of Arrowsmith et al. is the only other approach utilizing BlazePose for classifying physiotherapy exercises [31]. However, there was no emphasis on evaluating exercise performance, and the ML models were not trained or tested on actual patients.

We propose a methodology focused on older adults and patients that balances simplicity and thoroughness. By separating the recognition and evaluation tasks and opting for a single webcam as the sensing device, we adopted a straightforward design approach. Considering individual patient variations, we designed a personalized system, customized to the patient’s needs. The reliability of BlazePose measurements garnered overall positive outcomes. Our exercise recognition system demonstrated excellent performance, achieving 100% accuracy on the test set for each class, and maintained effectiveness in real-time experiments utilizing cameras with lower resolutions. Drawing upon the insights from our comprehensive analysis, we have successfully developed a cutting-edge TE system, primarily focusing on exercise recognition and evaluation. Furthermore, we integrated several advanced functionalities to offer a user-friendly environment. These features facilitate the monitoring of users’ activities and aim to control their responses. We envision our system being used either without supervision or as a complementary tool in videoconference mode with a trainer to monitor the trainee’s performance. Our TE system has the potential to serve as a valuable tool, offering motivation to patients and older adults throughout their home-based workout routines. To the best of our knowledge, even though the proposed tool is still under evaluation and improvement, this is the first study that incorporates and combines the aforementioned methods in a holistic, elderly-friendly TE system using BlazePose.

Our research is focused on demonstrating the potential of a real-time TE system based on our proposed methods. Future directions for this work should include enrichment with more exercises and records, testing the ML models on more patients, gamification for user engagement [32], audio feedback, voice command recognition for usability, and portability on devices like Raspberry Pi. Additionally, we consider incorporating fall detection algorithms to ensure patients’ safety. Finally, we could explore leveraging time series feature extraction libraries (TSFEL) [33] and linear discriminant analysis (LDA) for further experimentation and feature extraction refinement. These improvements in the maturation of the whole application should be followed by more extensive testing with users, especially older adults, to assess the acceptance, usability, and benefit of the solution. 

## 5. Conclusions

This work proposes an approach for monitoring physical exercise and providing guidance based on BlazePose motion recognition, the consequent analysis, and ML modeling of multiple traces. The main methodological components, kinematic analysis, and exercise recognition were successfully evaluated with real-life data, and an end-to-end procedure was proposed and demonstrated, showing the potential of the proposed system.

## Figures and Tables

**Table 1:** Guidelines and range of motion (ROM) analysis per exercise.

Name	Instructions	ROM Evaluation
SS	Take short, comfortable steps while ensuring an upright spinal posture.	Lateral ROM for both the right and left hips and comparison
SA	Maintain extended arms while ensuring an upright spinal posture.	ROM for both the right and left shoulders and comparison
TR	Maintain a shoulder-width stance, ensuring the arms are vertical to the body axis and keeping an upright spinal posture.	Lumbar rotation ROM for both sides and movement coordination analysis
FB	Maintain an upright spinal posture and leg extension when bending.	ROM involved in lumbar forward flexion
SQT	Maintain a shoulder-width stance, an upright spinal posture, and position the knees behind the toes.	Hip and knee flexion ROM

SS: Side steps, SA: shoulder abductions, TR: trunk rotation, FB: forward bends, SQT: squats, ROM: range of motion.

**Table 2:** Classifier comparison based on the performance metrics, calculated on the test set. The highest metrics are in bold and green and the lowest metrics are in red.

	285D Feature Vector							22D PCA Feature Vector						
	acc	F1	prec	sens	spec	TT	IT	acc	F1	prec	sens	spec	TT	IT
	[%]					[sec]		[%]					[sec]	
RF	**100**	**100**	**100**	**100**	**100**	**1.649**	**0.063**	99.63	100	100	100	100	1.205	0.054
${SVM}_{L}$	98.91	99	99	98.82	100	0.175	0.038	**100**	**100**	**100**	**100**	**100**	**0.105**	**0.029**
${SVM}_{P}$	95.12	95	95	95.69	100	0.842	0.108	**94.76**	**94**	**96**	**92.60**	**100**	**0.108**	**0.023**
${SVM}_{RBF}$	94.40	94	95	94.53	100	0.232	0.172	99.63	100	99	99.70	100	0.085	0.048
DT	**92.05**	**91**	**90**	**91.64**	**100**	**0.208**	**0.010**	97.29	98	98	97.69	100	0.250	0.001

acc: Accuracy, F1: F1 score, prec: precision, sens: sensitivity, TT: training time, IT: inference time, D: dimensional.

## Data Availability

The datasets generated or analyzed during the current study are available from the corresponding author upon reasonable request.

## Appendix A

The precise anthropometric measurements for the study population are outlined in Table A1. The average age of the patient group was 72.9 ± 12.7 years and ranged from 46 to 90 years, while for the trainers group it was 22.5 ± 8 years and ranged from 19 to 60 years. The average height and weight of male participants was 175.9 ± 5.1 cm and 72 ± 8.6 kg, while for female participants it was 165.6 ± 4.7 cm and 57.1 ± 6.7 kg.

The MP group was slightly younger (67 ± 14.9) than the FP group (77.8 ± 9.1), but no significant difference existed between the groups (*p* = 0.17). The FT group (21.5 ± 6.1) appeared younger than the MT group (24.4 ± 10.6), but no significant difference existed between the groups (*p* = 0.25). The male group was taller than the female group with a statistically significant difference (*p* = 0). A significant difference (*p* = 0) was also observed between the height of the FP group (161.3 ± 6.2 cm) and the FT group (166.5 ± 3.9 cm). FT were taller than FP. Weight significantly different between sexes (*p* = 0). A statistically significant difference (*p* = 0) was also observed between the weight of the MP group (80.6 ± 10.7 kg) and the MT group (69.5 ± 6.3 kg). Finally, a statistically significant difference (*p* = 0) was also observed between the weight of the FP group (65.8 ± 10.5 kg) and the FT group (55.3 ± 4 kg). Both MP and FP had higher weights compared to the trainers.

**Table A1:** Statistical analysis of the study population (n = 57).

Anthropometric Factors	Females (Mean ± SD)		Males (Mean ± SD)	
	FT	FP	MT	MP
Age [years]	21.5 ± 6.1	77.8 ± 9.1	24.4 ± 10.6	67 ± 14.9
Height [cm]	166.5 ± 3.9	161.3 ± 6.2	176.5 ± 4.9	173.8 ± 5.6
Weight [kg]	55.3 ± 4	65.8 ± 10.5	69.5 ± 6.3	80.6 ± 10.7

SD: Standard deviation.

## Appendix B

After a three-month interval, we re-examined three of the patients. As shown in Table A2, significant progress was observed in their performance. Through diligent monitoring, each individual demonstrated notable advancements in their ability to engage in physical activities. Only one person among the patients exhibited an augmentation in ROM across all exercises. This patient was a 46-year-old male who was experiencing paralysis on the right side of his body. He attended the rehabilitation program for 6 months, aiming to recover from a recent stroke. Conversely, for the other two patients, the progress of their performances varied. Importantly, even in cases where a decrease in ROM was documented, the detected execution errors were reduced. However, it should be noted that minor deviations in performance can be attributed to factors such as insufficient sleep, fatigue, stress, potential discomfort or pain [34], the presence or absence of a proper warm-up routine (known to enhance overall performance up to 79% [35]), and even exercise’s acceptable variations. In the pursuit of accurate evaluations, it becomes imperative to account for these variables to draw comprehensive conclusions about the effectiveness of the rehabilitation program.

**Table A2:** Progress of three patients after a three month re-examination.

	FP_1					FP_2					MP_3				
	SS	SA	TR	FB	SQT	SS	SA	TR	FB	SQT	SS	SA	TR	FB	SQT
ROM [°]	3.10	4.09	−6.36	−9.06	30.47	0	−3.35	27.16	−9.74	22.18	1.86	6.37	31.05	1.41	7.64
EE [%]	−1.99	2.15	−24.78	8.54	−3.71	−0.83	−9.36	−7.05	0	7.58	−8.39	1.42	−1.83	4.12	0

EE: Execution error, FP_x: female patient with the code x, MP_3: male patient with the code 3.

In conclusion, ΚA of the three-month retest recordings showed an increase in ROM up to 31.05° and a reduction in execution error up to 24.78% among patients. The observed progress not only underscores the positive impact on individual well-being but also highlights the importance of personalized care in enhancing physical activity and overall health outcomes for patients.
